# The total burden of rare, non-synonymous exome genetic variants is not associated with childhood or late-life cognitive ability

**DOI:** 10.1098/rspb.2014.0117

**Published:** 2014-04-22

**Authors:** Riccardo E. Marioni, Lars Penke, Gail Davies, Jennifer E. Huffman, Caroline Hayward, Ian J. Deary

**Affiliations:** 1 Centre for Cognitive Ageing and Cognitive Epidemiology, University of Edinburgh, 7 George Square, Edinburgh EH8 9JZ, UK; 2 Department of Psychology, University of Edinburgh, 7 George Square, Edinburgh EH8 9JZ, UK; 3 Institute of Psychology, Georg August University Göttingen, Goßlerstr. 14, Göttingen 37073, Germany; 4 Medical Genetics Section, Centre for Genomics and Experimental Medicine, Institute of Genetics and Molecular Medicine, University of Edinburgh, Edinburgh EH4 2XU, UK; 5 Medical Research Council Human Genetics Unit, Institute of Genetics and Molecular Medicine, University of Edinburgh, Edinburgh EH4 2XU, UK

**Keywords:** genetic burden, exome, general cognitive ability, intelligence, fitness, mutation–selection balance

## Abstract

Human cognitive ability shows consistent, positive associations with fitness components across the life-course. Underlying genetic variation should therefore be depleted by selection, which is not observed. Genetic variation in general cognitive ability (intelligence) could be maintained by a mutation–selection balance, with rare variants contributing to its genetic architecture. This study examines the association between the total number of rare stop-gain/loss, splice and missense exonic variants and cognitive ability in childhood and old age in the same individuals. Exome array data were obtained in the Lothian Birth Cohorts of 1921 and 1936 (combined *N* = 1596). General cognitive ability was assessed at age 11 years and in late life (79 and 70 years, respectively) and was modelled against the total number of stop-gain/loss, splice, and missense exonic variants, with minor allele frequency less than or equal to 0.01, using linear regression adjusted for age and sex. In both cohorts and in both the childhood and late-life models, there were no significant associations between rare variant burden in the exome and cognitive ability that survived correction for multiple testing. Contrary to our *a priori* hypothesis, we observed no evidence for an association between the total number of rare exonic variants and either childhood cognitive ability or late-life cognitive ability.

## 1. Introduction

Selection is expected to deplete genetic variation in traits over time [1]. This makes the existence of standing genetic variation puzzling in heritable traits that are consistently associated with fitness components [2]. Individual differences in various human cognitive abilities tend to be strongly correlated with each other, allowing the extraction of a single latent dimension that usually explains about 50% of the variation in broad batteries of cognitive tasks. This dimension is known as human general cognitive ability or general intelligence, conventionally abbreviated as *g* [3–5]. Twin, family and adoption studies consistently show that *g* is highly heritable in adulthood (50–80%) [6]. At the same time, *g* shows consistent, positive associations with fitness components such as lower mortality rate [7], better physical and mental health throughout the lifespan [8,9], height [10], developmental stability [11,12], sperm quality [13] and higher social status and resource acquisition potential, including better educational [14] and occupational success [15]. Furthermore, higher *g* is preferred in human mate choice [16–18]. This makes it unlikely that *g* adheres to the strict criteria for selective neutrality and suggests that its current genetic variation has been affected by directional selection, probably even more so historically, before the demographic transition [2,19,20]. Genetic variation can be maintained under directional selection in complex, dimensional traits such as cognitive ability when new, mostly deleterious mutations affecting the trait occur at a rate equal to how quickly they can be removed by selection, a state called mutation–selection balance [2]. Various estimation methods, including those based on genome-wide sequencing of parent–offspring trios, concur with high human genome-wide mutation rates [21]. Cognitive ability is not localized to a specific region of the brain, but instead is affected by extensive neural networks and brain-wide neuronal integrity [4,22,23]. Furthermore, associations with distal phenotypic measures such as fluctuating asymmetry of the body and face [11,12,24], as well as sperm quality [13], suggest that it is an indicator of overall system integrity or phenotypic condition [25]. These lines of evidence imply that harmful mutations in many parts of the genome might be able to affect cognitive ability [26], i.e. via a large mutational target size [27].

Since selection will quickly purge genetic variants with strong effects on fitness-related traits, genetic variation maintained by mutation–selection balance suggests a genetic architecture that lacks common genetic variants of notable effect sizes [2,28]. This is in line with the lack of replicable findings from candidate gene studies of cognitive ability [29] and the results from recent genome-wide association studies (GWAS) that suggest no individual common single nucleotide polymorphism (SNP) in the human genome explains more than 0.24% of the genetic variation [30]. However, genome-wide complex trait analyses (GCTA), which estimate cryptic distant genetic relationships in population samples from genome-wide SNP data, suggest heritabilities for *g* in the range of 30–50% [31]. Since these estimates are purely based on genome-wide common SNPs, they imply that a large part of the genetic architecture of *g* consists either of common genetic variants with individually miniscule phenotypic effects, or of rare genetic variants that have been in the population for long enough that they are in sufficient linkage disequilibrium with the common SNP markers present on commercial genotyping arrays. More recent mutations, including family-specific and private de novo genetic variants, could explain the discrepancy in heritability estimates from GCTA compared to those from twin, family and adoption studies [32].

A genetic architecture based on rare genetic variants implies high genetic heterogeneity [26,28] and makes extremely large samples necessary for genome-wide scans in order to detect individual causal variants, even if they have moderate-to-large effect sizes [32]. A different approach which is less affected by statistical power issues is to consider the total number of rare variants that an individual carries and then relate this to phenotypic traits. This approach relies on the assumption that it is the overall burden of deleterious mutations that should explain genetic variation in traits such as *g*, while the individual causal variants might be heterogeneous and interchangeable [26]. Using an estimate of genomic burden of rare copy number variants (CNVs), Yeo *et al*. [33] found a negative association between mutation burden and cognitive ability in a small sample of 74 individuals with alcohol dependence. However, three replication studies in much larger samples, using various estimates of genome-wide CNV burden, were unable to replicate this finding [34–36]. A different approach to mutation load is to look at the overall burden of rare (minor allele frequency, MAF ≤ 0.01) SNPs in the exome. To our knowledge, no study has yet looked at rare exomic SNP burden in relation to *g*. The recently developed exome array (http://genome.sph.umich.edu/wiki/Exome_Chip_Design), which genotypes rare variants identified from the exome sequencing of around 12 000 persons, provides an ideal resource to test this hypothesis.

While the rank-order stability of cognitive ability is high even from childhood to old age [37,38], cognitive ability does undergo differential age-related changes [39,40]. It is unclear whether the detrimental effects of exonic mutations affect cognitive ability equally across the lifespan, or alternatively more in the pre-reproductive life stage (childhood), when selection pressures against them should be stronger but higher developmental plasticity should provide a better buffer, or in the post-reproductive life stage (old age), when plasticity is low but selection pressures are assumed to be less effective against detrimental genetic effects [41].

The aim of this study was to examine the relationship between an individual's total number of rare exonic variants (SNPs with MAF ≤ 0.01) with cognitive ability measured in the same individuals at two points in life, during childhood and old age.

## 2. Methods

### (a) Population

Data stem from two Scottish birth cohorts: the Lothian Birth Cohorts (LBC) of 1921 (LBC1921) and 1936 (LBC1936), which are longitudinal studies of ageing [42–44].

LBC1921 participants were born in 1921 and most completed the Scottish Mental Survey 1932 (SMS1932) at mean age 11 years [45]. The SMS1932 assessment was administered nationwide to all 1921-born children who attended school in Scotland on 1 June 1932 and included the Moray House Test no. 12, which provides a measure of general cognitive ability. They were therefore tested at a mean age of 11 years. The LBC1921 attempted to follow up relatively healthy individuals who completed the SMS1932 in the Lothian region of Scotland (the area around Edinburgh city); 550 people were successfully traced and participated in the study from mean age 79 years. At present, there have been four waves of follow-up at mean ages 79, 83, 87 and 90 years where extensive cognitive, lifestyle, biomarker, neuroimaging, genetic and epigenetic data have been collected [42].

The design of LBC1936 is similar to LBC1921. Participants were born in 1936 and completed the SMS1947, which was administered nationwide to all children born in that year who attended school in Scotland on 4 June 1947 [46]. The Moray House Test no. 12 was used to assess cognitive ability. The LBC1936 follows up 1091 relatively healthy survivors from the SMS1947 who were living in the Lothian area at age about 70 years. Additional waves of data collection have been carried out at mean ages 73 and 76 years. As with LBC1921, extensive cognitive, lifestyle, biomarker, neuroimaging, genetic and epigenetic data have been collected across waves [42].

### (b) Ethics

Following informed consent, venesected whole blood was collected for DNA extraction in both LBC1921 and LBC1936. Ethics permission for the LBC1921 was obtained from the Lothian Research Ethics Committee (wave 1: LREC/1998/4/183; wave 2: LREC/2003/7/23; wave 3: 1702/98/4/183) and the Scotland A Research Ethics Committee (wave 4: 10/S1103/6). Ethics permission for the LBC1936 was obtained from the Multi-Centre Research Ethics Committee for Scotland (wave 1: MREC/01/0/56), the Lothian Research Ethics Committee (wave 1: LREC/2003/2/29) and the Scotland A Research Ethics Committee (waves 2 and 3: 07/MRE00/58). Written informed consent was obtained from all subjects.

### (c) Cognitive ability measures

For the current analysis, we consider two measures of cognitive ability. The same measure of general cognitive ability, the Moray House Test [45,46], was administered in childhood (age 11) and later life (age 79 in LBC1921, age 70 in LBC1936). It has a scoring range between 0 and 76. In addition, in late life, a principal components analysis was used to derive a fluid general cognitive ability factor for each cohort from the following cognitive test batteries: in LBC1921, the fluid cognitive tests included Raven's standard progressive matrices [47], verbal fluency [48] and Wechsler logical memory [49]; in LBC1936, the test battery included six tests from the Wechsler Adult Intelligence Scale-III UK (WAIS-III UK) [50]: digit symbol coding, block design, matrix reasoning, digit span backwards, symbol search and letter–number sequencing. *z*-standardized scores from the first principal component, which follow a Gaussian distribution with mean 0, variance 1, were saved and used as the measure of late-life cognitive ability.

### (d) Exome chip data

Genotype data were collected on the Illumina HumanExome genotyping array. Initial quality control was performed according to the CHARGE consortium criteria [51], leaving a total of 237 603 SNPs for analysis in 508 LBC1921 participants and 988 LBC1936 participants. A second stage of quality control was then conducted in Plink [52,53]. Firstly, haploid heterozygous calls were set to missing (*n* = 3590 in LBC1921, *n* = 8248 in LBC1936). Monomorphic SNPs (*n* = 164 711 in LBC1921, *n* = 154 692 in LBC1936), SNPs with a call rate less than 97% (*n* = 76 in LBC1921, 66 in LBC1936), and individuals with a call rate less than 97% (*n* = 8 in LBC1921, 1 in LBC1936) were removed. This left a total of 72 016 SNPs for downstream analysis in LBC1921 and 82 845 SNPs in LBC1936.

### (e) Measure of global burden

From the cleaned exome array data, stop-gain/loss, splice and missense SNPs with a MAF of less than or equal to 0.01 were extracted (*n* = 36 200 in LBC1921, *n* = 46 384 in LBC1936). The global burden of rare variants for each individual was defined as the total number of rare variant alleles (an additive model).

### (f) Statistical analysis

Linear regression models were built to assess the relationship between rare variant burden and childhood and late-life cognition in LBC1921 and LBC1936. Mean-centred age at cognitive assessment and sex were included as covariates in each model. The global burden of rare variants followed a Gaussian distribution; however, a few extreme outliers were removed prior to analysis (*n* = 2 in LBC1921, *n* = 3 in LBC1936). All models were re-run as part of a sensitivity analysis that used a less stringent cut-off for rare variants (MAF ≤ 0.05, *n*_SNPs_ = 44 045 in LBC1921 and *n*_SNPs_ = 54 439 in LBC1936). Models were also run at both the 1% and 5% MAF threshold to analyse individually the effects of rare variant burden on cognition for (i) stop-gain/loss, (ii) splice, (iii) missense and (iv) all variants, including synonymous SNPs. Diagnostic plots were examined to check model fit. All analyses were conducted in R [54]. Analysis code and a data file containing anonymized age, sex, cognitive test scores, and global burden data but not raw genetic data are available from the corresponding author on request.

## 3. Results

Descriptive data are presented in table 1. Owing to missing cognitive data there were slight differences in sample sizes in childhood and late life. At age 11, cognitive data were available on 446 LBC1921 and 940 LBC1936 participants. In late life these increased to 492 and 987, respectively. The mean ages of the samples in childhood were 10.9 years (s.d., 0.29) in LBC1921 and 10.9 years (s.d., 0.28) in LBC1936; in late life the mean ages of the samples were 79.0 (s.d., 0.58) and 69.6 (s.d., 0.84), respectively. The gender distribution was 59% female in LBC1921 and 50% female in LBC1936.

**Table 1.** Descriptive summary of LBC1921 and LBC1936 data in both childhood and late life. MAF, minor allele frequency.

	mean	s.d.
LBC1921 (*n* = 497, 59% female)		
childhood age (years)	10.9	0.29
late-life age (years)	79.0	0.58
Moray House Test no. 12 (age 11) (*n* = 446)	46.8	12.0
Moray House Test no. 12 (age 79) (*n* = 492)	59.4	11.0
fluid general cognitive ability, *g*^a^ (*n* = 486)	0.0	1.0
total number of rare alleles (MAF ≤ 0.01)	185.9	16.7
total number of rare alleles (MAF ≤ 0.05)	562.4	28.1
LBC1936 (*n* = 988, 50% female)		
childhood age (years)	10.9	0.28
late-life age (years)	69.6	0.84
Moray House Test no. 12 (age 11) (*n* = 940)	49.0	11.8
Moray House Test no. 12 (age 79) (*n* = 987)	64.3	8.8
fluid general cognitive ability, *g*^a^ (*n* = 983)	0.0	1.0
total number of rare alleles (MAF ≤ 0.01)	184.7	16.7
total number of rare alleles (MAF ≤ 0.05)	560.9	28.3

^a^Standardized variables, derived from principal components analysis.

The mean Moray House Test scores in childhood were 46.8 (s.d., 12.0) and 49.0 (s.d., 11.8) in LBC1921 and LBC1936, respectively. The corresponding values in late life were 59.4 (s.d., 11.0) and 64.3 (s.d., 8.8). The mean numbers of rare variants per individual were 185.9 (s.d., 16.7) in LBC1921 and 184.7 (s.d., 16.7) in LBC1936.

Table 2 presents four regression models of burden against general cognitive ability with adjustment for age and sex. There were no significant associations between burden of rare alleles and age-11 Moray House Test scores (LBC1921: *b* = −0.047 (s.e., 0.034), *p* = 0.17; LBC1936: *b* = 1.2 × 10^−3^ (s.e., 0.023), *p* = 0.96). Similar null findings were observed between burden and late-life Moray House Test scores (LBC1921: *b* = −8.4 × 10^−3^ (s.e., 0.030), *p* = 0.78; LBC1936: *b* = −0.016 (s.e., 0.017), *p* = 0.35). There were also null findings for the late-life models of burden against fluid general cognitive ability (LBC1921: *b* = −1.4 × 10^−3^ (s.e., 2.7 × 10^−3^), *p* = 0.62; LBC1936: *b* = 2.1 × 10^−3^ (s.e., 1.8 × 10^−3^), *p* = 0.26), which are presented in table 3.

**Table 2.** Age and sex adjusted linear regressions of burden (sum of variants with minor allele frequency less than or equal to 0.01) against childhood and late-life general cognitive ability (Moray House Test). LBC, Lothian Birth Cohort; MHT, Moray House Test scores; b, unstandardized beta; s.e., standard error; *p*, *p*-value.

	*b*	s.e.	*p*	*b*	s.e.	*p*
	LBC1921 childhood MHT			LBC1921 late-life MHT		
intercept	55.42	6.38	<0.001	62.63	5.54	<0.001
age (years)	7.97	1.95	<0.001	−1.19	0.86	0.16
sex (female)	0.23	1.13	0.84	−2.84	1.00	0.005
burden	−0.047	0.034	0.17	−8.4 × 10^−3^	0.030	0.78
	LBC1936 childhood MHT			LBC1936 late-life MHT		
intercept	48.38	4.22	<0.001	67.38	3.11	<0.001
age (years)	7.48	1.36	<0.001	−1.47	0.33	<0.001
sex (female)	1.75	0.76	0.021	−0.41	0.56	0.46
burden	−1.2 × 10^−3^	0.023	0.96	−0.016	0.017	0.35

**Table 3.** Age and sex adjusted linear regressions of burden (sum of variants with minor allele frequency ≤ 0.01) against late-life fluid cognitive ability factors. LBC, Lothian Birth Cohort; *g*, fluid general cognitive ability; *b*, unstandardized beta; s.e., standard error; *p*, *p*-value.

	LBC1921 late-life *g*			LBC1936 late-life *g*		
	*b*	s.e.	*p*	*b*	s.e.	*p*
intercept	0.43	0.51	0.40	−0.36	0.34	0.30
age (years)	−0.14	0.079	0.075	−0.32	0.037	<0.001
sex (female)	−0.27	0.093	0.004	−0.057	0.061	0.35
burden	−1.4 × 10^−3^	2.7 × 10^−3^	0.62	2.1 × 10^−3^	1.8 × 10^−3^	0.26

Some age and sex effects were observed in both early and late life and for both general and fluid cognitive ability (tables 2 and 3). Females were more likely to have slightly higher Moray House Test scores at age 11 (*b* = 0.23, *p* = 0.84 in LBC1921, *b* = 1.75, *p* = 0.021 in LBC1936) and slightly lower scores in later life (*b* = −2.84, *p* = 0.005 in LBC1921, *b* = −0.41, *p* = 0.46 in LBC1936). A similar pattern was seen for the late-life fluid abilities (*b* = −0.27, *p* = 0.004 in LBC1921, *b* = −0.057, *p* = 0.35 in LBC1936). Older children performed better on the Moray House Test at age 11 by around eight points per year (*p* < 0.001) but older adults had lower Moray House Test (*b* = −1.20, *p* = 0.16 in LBC1921, *b* = −1.47, *p* < 0.001 in LBC1936) and *g* scores (*b* = −0.14, *p* = 0.075 in LBC1921, *b* = −0.32, *p* < 0.001 in LBC1936) in late life.

Sensitivity analyses that included SNPs with a MAF less than 0.05 yielded near identical null associations to the models with a MAF cut-off of 0.01 (electronic supplementary material, tables S1 and S2). There were no associations between burden and cognitive ability for the models that considered (i) stop-gain/loss, (ii) splice, (iii) missense and (iv) all variants, including synonymous SNPs (electronic supplementary material, tables S3–S6). The only exception was for the splice variants in LBC1936, where a unit increase in rare variant burden decreased the childhood and late-life MHT scores by 0.3 points (*p* = 0.039 and 0.011, respectively). However, these associations were not significant after a Bonferroni correction to account for multiple testing.

## 4. Discussion

In this study, we found no evidence of a significant association for an individual's total number of rare exonic single nucleotide variants with childhood or late-life cognitive ability. Null associations were observed in all analyses after correction for multiple testing, suggesting that there is no link of total number of rare exonic stop-gain/loss, splice and missense SNPs with cognitive ability across the life-course in these two samples.

To our knowledge, this is the first time that a global sum score of rare exonic variants, representing mutation load, has been modelled with cognitive ability, a trait that correlates highly with several fitness components from across the life-course [7–14]. A unique feature of the LBCs makes it possible to investigate rare variant burden with cognitive measures from both childhood and late life. Unlike genome-wide studies of individual rare variants, or gene-based burden tests, this study is not hampered by statistical power issues and multiple testing of large numbers of genetic variants. However, our analysis is not designed to, nor is it able to, identify pathways that may link rare variants to cognitive ability.

One limitation of our analysis may be the lack of coverage on the exome array. Although over 36 000 rare SNPs were examined in each cohort, this does not include all rare variants that would be typed via whole-genome or whole-exome sequencing. Furthermore, the minor allele cut-off point for rare variants of 0.01 may have been too low (conservative) for the present analysis although re-adjusting this to 0.05 in a secondary analysis made no difference in the findings.

A possible explanation for our null finding might be that rare non-synonymous exonic SNPs, which make up the bulk (approx. 96%) of our variants, are not the right place to look for genetic variants explaining normal variation in cognitive ability. Non-synonymous exonic variants alter protein structures, which might result in too radical phenotypic effects to be observed in the normal, healthy population range. A sensitivity analysis that considered the burden of both synonymous and stop-gain/loss, splice and missense variants made very little difference to the association between total burden score cognitive ability. Exonic point mutations have been found to be mostly recent [55], which would be expected if they are strongly selected against for their large negative effects on fitness. Interestingly, Rauch *et al*. [56] found de novo point mutations were overrepresented in patients with severe non-syndromic intellectual disability (the low end of the cognitive ability continuum) compared with controls in an exome sequencing study and identified several loss-of-function exonic variants associated with intellectual disability. This contrasts with our study, which was not able to find similar effects in the normal, healthy range of cognitive ability differences. Indeed, the limited available evidence on the influence of de novo mutations on normal-range cognitive ability, stemming from genome-sequencing studies of parent–offspring trios [57] and associations of cognitive ability with paternal age [58–60], an indirect marker for de novo mutation load [61,62], suggests no such effects. This might be because de novo mutations, which have not experienced counteracting evolutionary selection yet, have mostly strong negative effects on cognitive ability and thus do not contribute to normal-range variation, and penetrant non-synonymous rare exonic variants might be overrepresented among them. Rare variants with more moderate effects that contribute to variation in normal cognitive ability might thus be more probably located in the intronic parts of the genome, and burden scores from whole-genome sequencing studies will thus be necessary to establish their effect. This would also imply that, in the normal range, non-synonymous mutational burden in intronic and exonic regions should be not or only weakly correlated, as it can be expected that the latter has much more severely negative phenotypic effects than the former. Against this background, it can be predicted that the overall burden of non-synonymous rare variants in intronic regions of the genome should negatively predict general cognitive ability in the normal range. Testing the mutation–selection balance model of intelligence this way will be possible in the near future based on whole-genome sequencing data. Finally, we did not examine potential gene×gene or gene×environment interactions.

Analyses such as the one conducted in this paper may also be better applied to complex traits with dichotomous end-points [26] e.g. Alzheimer's disease (AD) versus no-AD. Although general cognition in childhood and late life is an excellent marker of future disease risk and other comorbidities, there is no set threshold beyond which impaired functioning can be classified. Moreover, the LBCs are based on relatively healthy, successfully ageing subjects with exclusions made for dementia or severe cognitive impairment at (late life) baseline. The relatively old age at study entry, especially in LBC1921, may also have a bearing on the findings, with low-IQ individuals and those with poorer quality genes, health and fitness having died prior to this point. Furthermore, the average childhood cognitive scores for the LBC samples are higher, and with lower standard deviations than those of the general population of 11 year olds that completed the Scottish Mental Surveys of 1932 and 1947 [63]. The mean childhood Moray House Test score for LBC1921 was 46.8 (s.d., 12.0) compared with a national average of 34.5 (s.d., 15.5) [63]. The corresponding mean score for LBC1936 was 49.0 (s.d., 11.8), compared with the national and an Edinburgh, Fife and Lothians averages of 36.7 (s.d., 16.1) and 40.3 (s.d., 15.5), respectively [63]. Such range restriction on both mutation load and intelligence might have reduced our power to find a significant result. To this point, the standard deviation we found for exomic mutation load in both of our samples (16.7) was somewhat less than the lower bound of the standard deviation of Keller & Miller [64] estimated for disruptive exomic mutation load in humans.

In conclusion, contrary to our *a priori* hypothesis, we observed no evidence for an association between the total number of rare exonic variants and cognitive ability in childhood and old age. Future studies await the availability of whole-genome sequencing data in order to test associations of rare variant burden in the whole genome. These studies should also consider additional measures of fitness to see whether our findings are more widely applicable to other traits that can be considered being under mutation–selection balance. Ideally, these analyses should be conducted on populations where a full spectrum of the fitness measure is represented or where there is a well-defined dichotomous split in the trait.
